# A highly acid-resistant novel strain of *Lactobacillus johnsonii* No. 1088 has antibacterial activity, including that against *Helicobacter pylori,* and inhibits gastrin-mediated acid production in mice

**DOI:** 10.1002/mbo3.252

**Published:** 2015-03-15

**Authors:** Yuji Aiba, Yasuhiro Nakano, Yasuhiro Koga, Kenji Takahashi, Yasuhiko Komatsu

**Affiliations:** 1 Laboratory for Infectious Diseases, Tokai University School of MedicineIsehara, Kanagawa, 259-1103, Japan; 2 Snowden Co., Ltd.Chiyoda-ku, Tokyo, 101-0032, Japan

**Keywords:** Gastroesophageal reflux disease, *Helicobacter pylori*, lactic acid bacteria, *Lactobacillus johnsonii*, probiotics

## Abstract

A novel strain of *Lactobacillus johnsonii* No. 1088 was isolated from the gastric juice of a healthy Japanese male volunteer, and characterized for its effectiveness in the stomach environment. *Lactobacillus johnsonii* No. 1088 was found to have the strongest acid resistance among several lactobacilli examined (>10% of cells survived at pH 1.0 after 2 h), and such a high acid resistance property was a specific characteristic of this strain of *L. johnsonii*. When cultured with various virulent bacteria, *L. johnsonii* No. 1088 inhibited the growth of *Helicobacter pylori*,*Escherichia coli* O-157, *Salmonella* Typhimurium, and *Clostridium difficile*, in which case its effectiveness was more potent than that of a type strain of *L. johnsonii,*JCM2012. In addition to its effect in vitro, *L. johnsonii* No. 1088 inhibited the growth of *H. pylori* in human intestinal microbiota-associated mice in both its live and lyophilized forms. Moreover, *L. johnsonii* No. 1088 suppressed gastric acid secretion in mice via decreasing the number of gastrin-positive cells in the stomach. These results taken together suggest that *L. johnsonii* No. 1088 is a unique lactobacillus having properties beneficial for supporting *H. pylori* eradication by triple therapy including the use of a proton pump inhibitor (PPI) and also for prophylaxis of gastroesophageal reflux disease possibly caused after *H. pylori* eradication as a side effect of PPI.

## Introduction

Recently, probiotics have become widely recognized as the way to regulate the intestinal bacterial environment and to confer heath benefit on the host. Although the World Health Organization defined probiotics as “live micro-organisms which when administered in adequate amounts confer a health benefit on the host” (Gilliland et al. 2001), the main benefit of probiotics is now recognized as its effect in the intestines as originally suggested by Metchnikoff (1908). Although lactobacillus and bifidobacterium are typical genera used as probiotics (Prasad et al. 1998), other bacteria belonging to enterococcus or bacillus are also used for this purpose (Franz et al. 2003; Bader et al. 2012). It is said that probiotics are also good to prevent or alleviate cancer, constipation, inflammatory diseases, etc. through improving the intestinal microbiota (Mercenier et al. 2003).

In addition to the benefits of probiotic bacteria in the intestines, some beneficial effects on the stomach have been reported recently. Among them is the anti-*Helicobacter pylori* activity of selected probiotic bacterial strains. Midolo et al. (1995) reported that some strains of lactic acid-producing bacteria inhibit the growth of *H. pylori* in vitro, where not only lactic acid but also other extracellular compounds produced by these bacteria were suggested to be anti-*H. pylori* agents. Kabir et al. (1997), using a gnotobiotic murine system, found that gnotobiotic mice bearing with *Lactobacillus salivarius* or lactobacilli that originated from murine stomach were largely protected from colonization by *H. pylori*, whereas *Enterococcus faecalis* and *Staphylococcus aureus* did not have such a preventive effect. Using a mouse model we confirmed the therapeutic effect of *L. salivarius* but not that of *Lactobacillus casei* or *Lactobacillus acidophilus*; that is, *L. salivarius* could eliminate or suppress *H. pylori* colonization when administered after a *H. pylori* infection (Aiba et al. 1998).

In clinical situations, *H. pylori* is eliminated by treatment with multiple antibiotics in combination with a proton pump inhibitor (PPI) (Malfertheiner et al. 2007). However, even after successful elimination of *H. pylori*, lowering of the pH in the stomach after *H. pylori* eradication is possibly problematic, resulting in gastroesophageal reflux disease (GERD), especially in Asian populations (Xie et al. 2013). Although some studies have not supported the negative relationship between eradication *H. pylori* and risk of GERD (McColl et al. 2000; Malfertheiner et al. 2002), prevention of excess gastric acid secretion might be beneficial to prevent GERD. Since we demonstrated that some species of lactobacilli even as heat-killed forms decreased the number of gastrin-positive cells in stomach and elevated the pH of gastric juice (Takahashi et al. 2011), appropriate species of lactobacilli are thought to be beneficial for both eradication of *H. pylori* and lowering the risk of GERD after *H. pylori* eradication.

In the present study we report the characteristics of a new strain of *Lactobacillus johnsonii* No. 1088, which was highly acid resistant and showed strong anti-*H. pylori* activity in mice. This strain also decreased the number of gastrin-positive cells and improved the acidic state of the stomach after *H. pylori* eradication in mice.

## Materials and Methods

### Bacterial strains

*Lactobacillus johnsonii* No. 1088 was isolated from gastric juice of a healthy Japanese male, and identified as *L. johnsonii* according to the method described in Bergey's Manual of Systematic Bacteriology vol. 2 and by comparing its genomic DNA sequence encoding 16S ribosomal RNA with that of type and reference strains (*L. johnsonii* ATCC33200 and *L. johnsonii* NCC533, respectively). This strain was deposited at the National Institute of Technology and Evolution (Chiba, Japan) as Accession No. NITE P-278. *Lactobacillus johnsonii* La1, *L. gasseri* OLL2716, *L. casei* shirota, *L. rhamnosus* GG, *L. acidophilus* BF, and *L. brevis* KB290 were isolated from commercially available fermented products. *Lactobacillus johnsonii* JCM2012, *L. gasseri* JCM1131, and *Clostridium difficile* JCM1296 were obtained from the Japan Collection of Microorganisms (RIKEN, Ibaraki, Japan). *Helicobacter pylori* No. 130, *Escherichia coli* O-157, *Salmonella* Typhimurium LT2, *Candida albicans* TI3001 were strains isolated at Tokai University Hospital.

### Resistance of lactobacilli to low pH

Lactobacilli were cultured in de Man, Rogosa and Sharpe (MRS) broth (BD, NJ, USA) for 18 h at 37°C to prepare growth-phase bacteria to be tested. The grown bacterial cultures were diluted with 0.1 mol/L HCl-citrate buffer (pH 2.0, 1.5 or 1.0) to about 10^7^ CFU/mL, and incubated at 37°C up to 120 min. The residual numbers of viable bacteria were determined at various time points.

### Mixed-culture study of *L. johnsonii* with other bacteria

*Lactobacillus johnsonii* No. 1088 or JCM2012 (10^6^ CFU/mL) was cocultured with *H. pylori* No. 130 (10^7^ CFU/mL) in Brain-Heart Infusion (BHI) broth (BD) supplemented with 5% horse serum at 37°C under microaerobic culture conditions up to 48 h, with the number of viable *H. pylori* being determined at various time points. As a control, *H. pylori* was cultured without lactobacilli. To examine the effect of *L. johnsonii* cocultured with other pathogenic bacteria instead of *H. pylori*, we cocultured *E. coli* O-157 (10^7^ CFU/mL), *S*. Typhimurium LT2 (10^7^ CFU/mL), *C. difficile* JCM1296 (10^7^ CFU/mL) or *C. albicans* TI3001 (10^6^ CFU/mL) with *L. johnsonii* No. 1088 or JCM2011 (10^6^ CFU/mL) in Gifu Anaerobic Medium (GAM) broth (Nissui, Tokyo, Japan) supplemented with 0.5% glucose, BHI broth, BHI broth (anaerobic condition), BHI broth, respectively, and then determined the numbers of viable bacteria at various time points.

### Anti-*H. pylori* activity of *L. johnsonii* No. 1088 in human intestinal microbiota-associated mice

All animal experiments reported in this study were carried out in accordance with the institutional guidelines of Tokai University. Male germ-free mice were purchased from Clea Japan, Inc. (Tokyo, Japan) and maintained in Trexler-type flexible-film plastic isolators with sterile food and water. At the age of 4 weeks, human feces obtained from a healthy Japanese male volunteer were orally administered to each animal (0.5 mL of about 10 mg/mL of feces diluted in phosphate buffer). After 4 weeks, *H. pylori* No. 130 (10^9^ CFU/mice) was orally administered to these animals four times (once a day for 4 days). After 11 days after the last administration of *H. pylori* (day 0), oral administration of *L. johnsonii* No. 1088 was started. For examination of the effect of culture broth of *L. johnsonii* No. 1088, six animals were sacrificed at day 0 to know the number of *H. pylori* before *L. johnsonii* treatment. Twenty animals were administered 0.5 mL of culture broth of *L. johnsonii* No. 1088 (about 10^9^ CFU) once a day. In another experiment, lyophilized powder of *L. johnsonii* No. 1088 containing the same number of bacteria was used instead of the culture broth. After 2 weeks (day 14), 10 animals were sacrificed; and then 2 weeks later (day 28), the remaining 10 animals were sacrificed to examine the number of *H. pylori* in the stomach. As controls, 10 animals without *L. johnsonii* No. 1088 administration were maintained under the same conditions and sacrificed at day 14, with another 10 killed at day 28.

### Gastric acid production in germ-free mice

Male germ-free Balb/c mice (8-weeks old) were administered 10^9^ CFU of *L. johnsonii* No. 1088 (*n* = 5) suspended in phosphate-buffered saline (PBS). The control group received PBS without bacteria (*n* = 5). After 10 days from the administration, mice were anesthetized with Nembutal, their stomach was exposed, and the pylorus and duodenum were clamped with forceps. After 2 h, gastric juice was collected and centrifuged at 3000*g* for 5 min, after which its volume, total acidity, and pH were determined. Total acidity was determined by titration with 0.1 N NaOH, and pH was measured with a pH meter (Horiba, Tokyo, Japan).

### Immunohistochemical analysis of gastrin-positive cells

**Figure 1:**
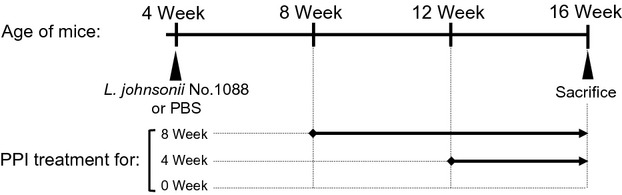
Schematic drawing of time schedule of the experiment to examine the effect of proton pump inhibitor (PPI) and *Lactobacillus johnsonii* No. 1088 on the number of gastrin-positive cells and stomach weight.

The number of gastrin-positive cells in the stomach of mice was examined by an immunohistochemical method described previously (Takahashi et al. 2011) to examine the effect of PPI and *L. johnsonii* No. 1088 on the number of gastrin-positive cells. As shown schematically in Figure1, at 8 weeks of age, s.c. administration of 200 *μ*g of the PPI omeprozaol every 2 days was started in 4–5 mice (8-week PPI treatment group). At 12 weeks of age, 3–5 other mice in each group were started on the PPI as above (4-week PPI treatment group). All mice were then sacrificed when 16 weeks old, and their stomach weight and number of gastrin-positive cells were determined. After being sacrificed, mice were weighed and their stomachs were resected. The resected stomachs were rinsed with PBS, weighed, and then fixed in 10% formalin buffered with PBS. The fixed tissues were embedded in paraffin and cut into 2-*μ*m-thick sections. The tissue sections were deparaffinized, microwaved for 10 min in Target Retrieval Solution (Dako, Glostrup, Denmark), and stained with rabbit polyclonal antigastrin antibody (Dako) as the primary antibody followed by goat anti-rabbit IgG labeled with Alexa488 (Molecular Probes, OR, USA) as the secondary antibody. Finally, the sections were stained with DAPI (4,6-diamidino-2-phenylinodole). The stained slides were observed with a fluorescence microscope (BZ-9000; Keyence, Osaka, Japan). AxioVision release 4.8 (Zeiss, Jena, Germany) was used to count the number of gastrin-positive cells in sections in random fields 1 mm in length along the corpus-antrum axis.

### Immunological quantification of serum gastrin concentration

The serum concentration of gastrin was determined by use of a radio immunoassay (Gastirn RIA kit II; TFB, Tokyo, Japan) according to the manufacturer's instruction at SRL (Tokyo, Japan).

### Statistics

Statistical significance between two groups was examined with Student's *t*-test. Comparison between multiple groups (>2) was performed either with Tukey's honestly significant difference test (for analyzing between all groups) or Dunnett's test (for analyzing between control and other groups).

## Results

### Isolation and identification of *L. johnsonii* No. 1088

To obtain extremely acid-resistant lactobacilli, we isolated lactobacilli from human gastric juice. Gastric juices (1 mL each) were sampled from 10 healthy human volunteers and used as the sources for bacterial isolation. The samples were variously diluted with sterile saline, cultured on BL (glucose-Blood-Liver; Nissui) or MRS (BD) agar under anaerobic conditions for 48 h at 37°C. The colonies that appeared were isolated and cultured in MRS broth (BD Biosciences). Colony picking and cultivation were repeated three times to obtain purified bacterial strains. Totally 15 strains were selected as potential lactobacilli (gram positive, rod shape, catalase negative, motility negative, sporogenesis negative, and lactic acid production positive). The strain used in this study was selected as the most acid resistant one among them, that is, giving the maximum number of viable bacteria after incubation for 120 min at 37°C at pH 2.0 (0.1 mol/L sodium citrate-hydrochloric acid buffer).

This strain was identified as *L. johnsonii* according to Bergeys' Manual of Systematic Bacteriology vol. 2, and further confirmed by comparing its genomic DNA sequence encoding 16S ribosomal RNA to that of type and reference strains (*L. johnsonii* ATCC33200 and *L. johnsonii* NCC533, respectively). The sequence was 99.7% and 100% identical to that of *L. johnsonii* ATCC33200 and *L. johnsonii* NCC533, respectively. To deny the possibility of contamination from a commercially available strain in yogurt (*L. johnsonii* La1), PCR products amplified by using several short primers (atgagagacg, gcacgcgaat, agcgagatgt, ctgccgattg, cgaggtcagt, or gtgagttgca) were compared (Williams et al. 1990) and found to be different from those of La1 (data not shown). We named this new strain as *L. johnsonii* No. 1088.

### Acid resistance of *L. johnsonii* No. 1088 and other lactobacilli

**Figure 2:**
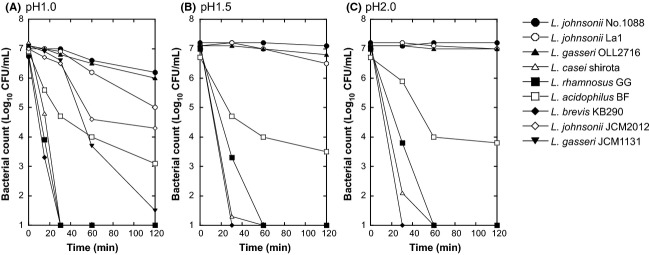
Viability of *Lactobacillus johnsonii* No. 1088 and other lactobacilli at pH 1.0 (A), 1.5 (B), and 2.0 (C). Growing bacteria (*L. johnsonii* No. 1088, *L. johnsonii* La1, *L. gasseri* OLL2716, *L. casei* shirora, *L. rhamnosus* GG, *L. acidophilus* BF, and *L. brevis* KB290 for pH 1.0, 1.5, and 2.0; and type strains *L. johnsonii* JCM2012 and *L. gasseri* JCM1131 added for pH 1.0) were diluted at a density around 10^7^ CFU/mL in acidic buffer and then incubated at 37°C up to 120 min. Counts of living bacterial counts were made at various time points.

Next we compared the viability of *L. johnsonii* No. 1088 in an acidic environment with that of other lactobacilli. Figure2 shows the viabilities of commercially available lactobacilli when incubated at pH 1.0 (Fig.2A), 1.5 (Fig.2B) or 2.0 (Fig.2C) at 37°C up to 120 min. Among the lactobacilli examined, *L. johnsonii* No. 1088, *L. johnsonii* La1, and *L. gasseri* OLL2716 were highly acid resistant (>1–10% were viable even after 120 min at pH 1.0), with *L. johnsonii* No. 1088 being the most. *Lactobacillus casei* shirota, *L. rhamnosus* GG, and *L. brevis* KB290 were dead within 30 min at pH 1.0, whereas *L. acidophilus* BF showed intermediate resistance to acid. Figure2A also shows the viabilities of type strains of *L. johnsonii* JCM2012 and *L. gasseri* JCM1131. As shown in this figure, the strong acid resistance property was strain specific rather than a common property of the respective species.

### Antibacterial effect of *L. johnsonii* No. 1088 in mixed cultures

**Figure 3:**
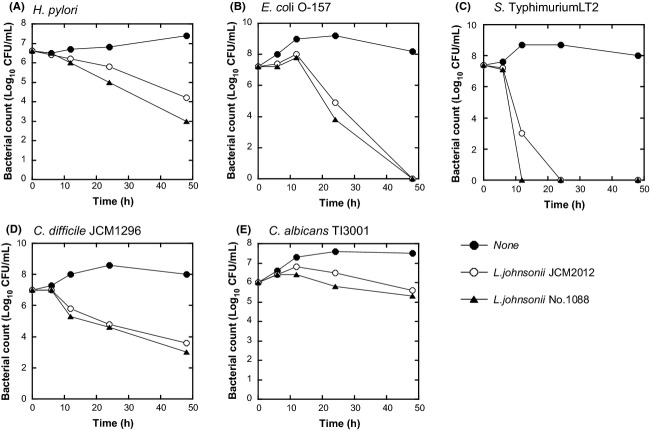
Antibacterial effect of *Lactobacillus johnsonii* No. 1088 and its type strain *L. johnsonii* JCM2012 under mixed-culture conditions. *Lactobacillus johnsonii* No. 1088 or JCM2012 (10^6^ CFU/mL) was co-cultured with various virulent bacteria (A, *Helicobacter pylori* No. 130 [10^7^ CFU/mL]; B, *Escherichia coli* O-157 [10^7^ CFU/mL]; C, *Salmonella*. Typhimurium LT2 [10^7^ CFU/mL]; D, *Clostridium difficile* JCM1296 [10^7^ CFU/mL] or E, *Candida albicans* TI3001 [10^6^ CFU/mL]), and numbers of viable bacteria were determined at the various time points indicated. Symbols are defined in the figure itself.

Since lactobacilli have been reported to have antimicrobial activity (Kailasapathy and Chin 2000), next we examined *L. johnsonii* for such an effect on various virulent bacteria. Figure3 shows the viabilities of various virulent bacteria when these bacteria were cocultured with *L. johnsonii* JCM2012 or No. 1088 up to 48 h. Although *H. pylori* No. 130 alone grew about threefold in 48 h, when co-cultured with *L. johnsonii* JCM2012 or No. 1088 the number of *H. pylori* decreased to about 1/300 or 1/3000, respectively (Fig.3A). The growth of *E. coli* O-157 (Fig.3B), *S*. Typhimurium LT2 (Fig.3C), and *C. difficile* JCM1296 (Fig.3D) was also strongly inhibited when these bacteria were co-cultured with *L. johnsonii* JCM2012 or No. 1088, in which case *L. johnsonii* No. 1088 was slightly more effective than JCM2012. The number of viable *C. albicans* TI3001 cells was not decreased when these cells were cocultured with *L. johnsonii* (Fig.3E), but their growth in cocultures was inhibited. These results suggest that *L. johnsonii* No. 1088 and JCM2012 were effective in suppressing the growth of various virulent bacteria and that such an antibacterial effect was stronger for No. 1088 than for JCM2012, especially against *H. pylori*.

### Anti-*H. pylori* activity of *L. johnsonii* No. 1088 in human intestinal microbiota-associated mice

**Figure 4:**
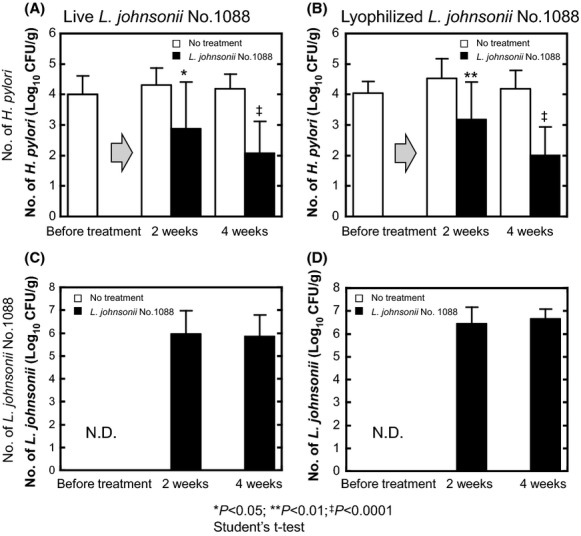
Anti-*Helicobacter pylori* effect of *Lactobacillus johnsonii* No. 1088 in human intestinal microbiota-associated mice. Mice associated with human intestinal microbiota were prepared by using germ-free mice and then infected with *Helicobacter pylori* No. 130 (10^9^ CFU/mice) as described in Materials and Methods. *Helicobacter pylori*-bearing mice were orally and daily administered growing live *L. johnsonii* No. 1088 (A and C) or a comparable number of lyophilized cells (B and D) for 2 or 4 weeks. In both mice treated with live or the lyophilized form of *L. johnsonii* No. 1088, the number of *H. pylori* in the stomach significantly decreased (A and B). Along with the decrease in the number of *H. pylori*, a steady number of *L. johnsonii* No. 1088 was detected in the stomach (C and D). Statistical significance was determined by use of Student's *t*-test (*P < 0.05, **P < 0.01, ^‡^*P* < 0.0001 versus no treatment for comparable time periods).

To confirm the anti-*H. pylori* activity of *L. johnsonii* No. 1088 found in vitro (Fig.3A), we examined its activity in vivo. Figure4 shows the effect of *L. johnsonii* No. 1088 on the number of *H. pylori* No. 130 in human intestinal microbiota-associated mice. As shown in Figure4A and B, *L. johnsonii* No. 1088 reduced the number of *H. pylori* in the stomach to about 1/100 after 4 weeks' oral administration; and *L. johnsonii* No. 1088 was effective when administered not only as culture broth (Fig.4A) but also as its lyophilized powder form (Fig.4B). Administration of either form of *L. johnsonii* No. 1088 resulted in living *L. johnsonii* No. 1088 in the stomach (Fig.4C and 4D).

### Effect of *L. johnsonii* No. 1088 on gastric acid production

Triple therapy including PPI is now widely used to eradicate *H. pylori* in clinical situations (Malfertheiner et al. 2007). But lowering of the gastric pH after successful eradication of *H. pylori* has been recognized as a potential risk factor of GERD (Xie et al. 2013). As demonstrated above, *L. johnsonii* No. 1088 was found useful for eradication of *H. pylori*. Next we examined whether this lactobacillus strain would also be beneficial for lowering the risk of GERD. Table1 shows volume, total acidity, and pH of gastric juice of germ-free Balb/c mice 10 days after having been administered 10^9^ CFU of *L. johnsonii* No. 1088. Although the volumes of gastric juice were the same irrespective of the administration of *L. johnsonii* No. 1088, the pH of the gastric juice was significantly (*P* < 0.005) higher when *L. johnsonii* No. 1088 was administered (pH 6.1) than when the control PBS was given (pH 3.0). Moreover, the total acidity in the *L. johnsonii* No. 1088-treated group (5.2 mEq/mL) tended to be lower than that in the control one (19.0 mEq/mL), although there was no statistically significant difference (*P* = 0.051).

**Table 1:** Volume, acid concentration, and pH of gastric juice after administration of *Lactobacillus johnsonii* No. 1088

Treatment	Volume of gastric juice (mL)	Acid concentration (mEq/mL)	pH	*n*
PBS	0.6 ± 0.2	19.0 ± 4.4	3.0 ± 0.5	5
*L. johnsonii* No. 1088	0.6 ± 0.3	5.2 ± 5.1*	6.1 ± 0.7**	5

PBS, phosphate-buffered saline.

Significance of difference versus PBS:

* *P = *0.051;

** *P *<* *0.005.

### Effect of *L. johnsonii* No. 1088 on PPI-induced increase in gastrin-positive cells and weight of stomach

**Figure 5:**
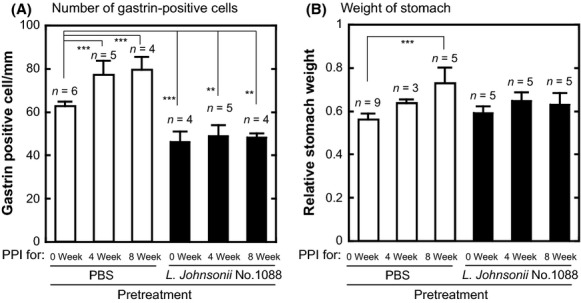
Effect of proton pump inhibitor (PPI) and *Lactobacillus johnsonii* No. 1088 on the number of gastrin-positive cells and stomach weight. Germ-free Balb/c mice (4 weeks old) were orally administered 10^9^ CFU of live *L. johnsonii* No. 1088 (closed bars) or PBS (open bars). At 8 weeks of age, 4–5 mice in each group were administered s.c. with 200 *μ*g of the PPI omeprozaol every 2 days (8-week PPI treatment group). At 12 weeks of age, the remaining 3–5 mice in each group were administered PPI as above (4-week PPI treatment group). At 16 weeks of age, all mice were sacrificed; and the number of gastrin-positive cells (A) and stomach weight (B) were analyzed as described in Materials and Methods. Statistical significance of difference between groups was determined by Tukey's honestly significant difference test and summarized in Table2, whereas selected results are indicated in the graph (**P < 0.01; ***P < 0.001 as indicated by the brackets).

To know the underlying mechanism, we next examined the effect of *L. johnsonii* No. 1088 on the number of gastrin-positive cells. Since gastrin is a hormone produced by G-cells in the pyloric antrum of the stomach and stimulates the secretion of gastric acid, it is interesting to know whether PPI treatment and administration of *L. johnsonii* No. 1088 would affect the number of these cells. Germ-free Balb/c mice (4 weeks old) were orally administered 10^9^ CFU of live *L. johnsonii* No. 1088 or PBS. The effects of treatment with PPI with and without *L. johnsonii* No. 1088 on the number of gastrin-positive cells and stomach weight are shown in Figure5A and B, respectively; and the statistical analysis of these data (Tukey's honestly significant difference test) are summarized in Table2. As shown in Figure5A, the administration of *L. johnsonii* No. 1088 decreased the number of gastrin-positive cells (compared with PPI for 0W; *P* = 0.0003), whereas the stomach weight was not different (Fig.5B). PPI administration increased both the number of gastrin-positive cells (Fig.5A, open bars) and stomach weight (Fig.5B, open bars). Since gastrin stimulates the growth of the fundic mucosa (Kinoshita and Ishihara 2000), this result can be explained by a probable increase in gastrin secretion. On the other hand, preadministration with *L. johnsonii* No. 1088 significantly prevented the increase in the number of gastrin-positive cells (Fig.5A, closed bars) and also that in stomach weight (Fig.5B, closed bars).

**Table 2:** *P*-values after determination of gastrin-positive cells/mm and stomach weight for Figure5

Test condition	PBS			*Lactobacillus johnsonii* No. 1088		
	0 Week	4 Weeks	8 Weeks	0 Week	4 Weeks	8 Weeks
(A) Gastrin-positive cells/mm						
PBS						
0 Week	–	–	–	–	–	–
4 Weeks	**0.0004**	–	–	–	–	–
8 Weeks	**0.0001**	0.9691	–	–	–	–
*L. johnsonii* No. 1088						
0 Week	**0.0003**	**<0.0001**	**<0.0001**	–	–	–
4 Weeks	**0.0012**	**<0.0001**	**<0.0001**	0.9501	–	–
8 Weeks	**0.0014**	**<0.0001**	**<0.0001**	0.9886	0.9999	–
(B) Relative stomach weight						
PBS						
0 Week	–	–	–	–	–	–
4 Weeks	0.102	–	–	–	–	–
8 Weeks	**<0.0001**	0.0604	–	–	–	–
*L. johnsonii* No. 1088						
0 Week	0.6342	0.7772	**0.0005**	–	–	–
4 Weeks	**0.0063**	0.9949	0.0809	0.3125	–	–
8 Weeks	**0.0426**	1	**0.0165**	0.725	0.9792	–

Statistical significances were determined by Tukey's honestly significant difference test. *P *<* *0.05 is indicated by bold letters. PBS, phosphate-buffered saline.

### Decrease in serum gastrin concentration by ingested heat-killed *L. johnsonii* No. 1088

**Figure 6:**
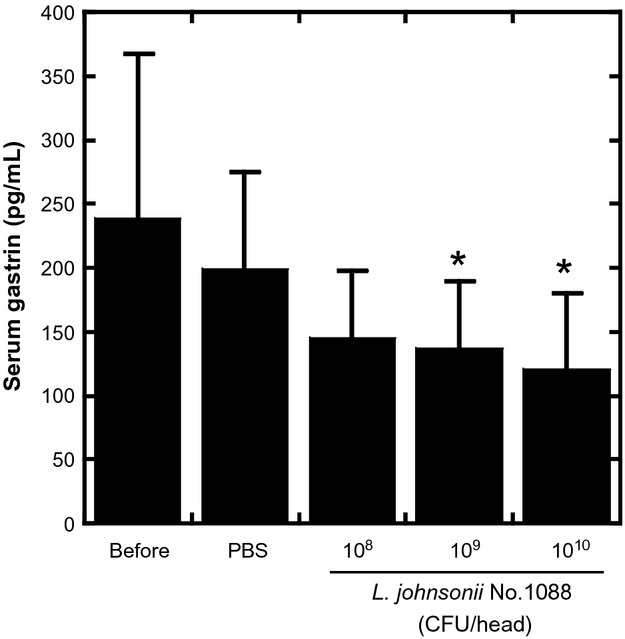
Decrease in serum gastrin concentration after oral treatment of germ-free mice with heat-killed *Lactobacillus johnsonii* No. 1088 for 10 days. Various numbers of heat-killed *L. johnsonii* No. 1088 were orally administered for 10 days, and serum concentrations of gastrin were determined with ELISA. Administration with 10^9^ or 10^10^ CFU of heat-killed *L. johnsonii* No. 1088 significantly decreased the serum gastrin concentration. *P < 0.05 versus PBS (Dunnett's test).

Finally, we examined the effect of oral administration of *L. johnsonii* No. 1088 on the serum concentration of gastrin. As shown in Figure6, oral administration of heat-killed *L. johnsonii* No. 1088 for 10 days to germ-free Balb/c mice dose dependently decreased their serum concentration of gastrin; the gastrin concentration was significantly lower at a dose of 10^9^ or 10^10^ CFU/mouse than that before treatment (*P* < 0.05).

## Discussion

Acid resistance has been recognized as one of the important factors for selecting probiotic strains of lactic acid bacteria, since survival during passage through the stomach is a key factor for the bacteria to reach the intestines alive. Although *L. acidophilus* and bifidobacteria have been reported to be resistant to acid, such a resistance is not intrinsic to the species but is highly dependent on the strain (Charteris et al. 1998; Kailasapathy and Chin 2000). In the present study, *L. johnsonii* No. 1088 was found to be in the group of the most acid-resistant lactobacilli. This group also contained *L. gasseri* OLL2716, which had earlier been selected from 203 strains of lactobacilli (Sakamoto et al. 2001). Since other strains of *L. johnsonii* and *L. gasseri* were not so resistant to acid (Fig.1A), such high acid resistance of *L. johnsonii* No. 1088 and *L. gasseri* OLL2716 may be considered to be specific to these strains. Although several mechanisms have been proposed to explain the acid resistance of gram-positive bacteria (Cotter and Hill 2003), the underlying mechanism of the high acid resistance of *L. johnsonii* No. 1088 remains to be elucidated.

*Lactobacillus acidophilus* and bifidobacteria have been reported to have wide-spectrum antipathogenic bacterial activity, including that against *S. aureus*,*S*. Typhimurium, *Yersinia enterocolitica*, and *Clostridium perfringens* (Kailasapathy and Chin 2000). Furthermore, anti-*H. pylori* activity of lactobacilli has been reported for several probiotic strains, including *L. acidophilus*,*L. casei*,*L. salivarius*,*L. reuteri*,*L. johnsonii*, and *L. gasseri* (Midolo et al. 1995; Kabir et al. 1997; Michetti et al. 1999; Felley et al. 2001; Francavilla et al. 2008; De Vuyst et al. 2010). Although competitive colonization and production of various compounds having microbial activities (organic acids, bacteriocins, hydrogen peroxide, carbon dioxide, etc.) have been proposed as the probable mechanisms underlying the antibacterial activity of lactobacilli (Kailasapathy and Chin 2000), the main mechanism of the anti-*H. pylori* activity of *L. johnsonii* No. 1088 has not yet been addressed. One of the possible anti-*H. pylori* agents might be peptides derived from *L. johnsonii* No. 1088 as suggested for anti-*H. pylori* activity of *L. johnsonii* La1 (De Vuyst et al. 2010). The clinical effectiveness of lactobacilli is controversial depending on the strains used for studies, but some promising results have been reported regarding *L. gasseri* OLL2716 (Sakamoto et al. 2001), *L. reuteri* (Francavilla et al. 2014), and *L. johnsonii* La1 (Felley et al. 2001). However, complete eradication of *H. pylori* solely by ingestion of lactobacilli cannot be attained. Instead, it is a promising strategy to use lactobacilli as a support for eradication with other therapies including multiple antibiotics. Since *L. johnsonii* No. 1088 was extremely acid resistant, a strong supportive effect of it for *H. pylori* eradication in clinical situations might be expected as observed in *L. gasseri* OLL2716 (Fujimura et al. 2006).

Another interesting characteristic of *L. johnsonii* No. 1088 was its ability to inhibit gastric acid production by decreasing the number of gastrin-positive cells in the stomach. In a previous study, we demonstrated that such an effect of *L. johnsonii* No. 1088 was slightly stronger than that of *L. gasseri* OLL2716 (Takahashi et al. 2011). In the present study, we demonstrated that *L. johnsonii* No. 1088 suppressed the gastrin production increased by PPI administration (Fig.5). These results clearly suggest that *L. johnsonii* No. 1088 should be useful for not only supporting *H. pylori* eradication therapy but also prevention of the risk of GERD after *H. pylori* eradication therapy including PPI. Although the mechanism by which *L. johnsonii* No. 1088 decreases the number of gastrin-positive cells is not clear to date, activation of Toll-like receptor 2 (TLR2) by cell surface components of *L. johnsonii* No. 1088 might be one candidate, since a known TLR2 ligand, pam3 (Takeda et al. 2002), but not TLR4 ligand, Lipopolysaccharide (LPS), reduced the number of gastrin-producing cells as well (Y. Nakano, unpubl. result). Further study will be necessary to confirm this hypothesis.

In conclusion, *L. johnsonii* No. 1088 was presently shown to be a unique lactobacillus, having an extremely acid resistance property, strong antibacterial activity including that against *H. pylori* and also inhibiting gastrin-induced acid production in germ-free mice. We propose the combination of these properties to be beneficial for supporting *H. pylori* eradication by antibiotics triple therapy including PPI, and also for prophylaxis of GERD possibly caused after *H. pylori* eradication as a side effect of PPI treatment.

## Conflict of Interest

Y. Aiba, Y. Nakano, K. Takahashi, and Y. Komatsu are employees of Snowden Co., Ltd.
